# AtFusionDB: a database of fusion transcripts in *Arabidopsis thaliana*

**DOI:** 10.1093/database/bay135

**Published:** 2019-01-08

**Authors:** Ajeet Singh, Shafaque Zahra, Durdam Das, Shailesh Kumar

**Affiliations:** Bioinformatics Laboratory, National Institute of Plant Genome Research Aruna Asaf Ali Marg, New Delhi, India

## Abstract

Fusion transcripts are chimeric RNAs generated as a result of fusion either at DNA or RNA level. These novel transcripts have been extensively studied in the case of human cancers but still remain underexamined in plants. In this study, we introduce the first plant-specific database of fusion transcripts named AtFusionDB (http://www.nipgr.res.in/AtFusionDB). This is a comprehensive database that contains the detailed information about fusion transcripts identified in model plant *Arabidopsis thaliana*. A total of 82 969 fusion transcript entries generated from 17 181 different genes of *A. thaliana* are available in this database. Apart from the basic information consisting of the Ensembl gene names, official gene name, tissue type, EricScore, fusion type, AtFusionDB ID and sample ID (e.g. Sequence Read Archive ID), additional information like UniProt, gene coordinates (together with the function of parental genes), junction sequence, expression level of both parent genes and fusion transcript may be of high utility to the user. Two different types of search modules *viz.* ‘Simple Search’ and ‘Advanced Search’ in addition to the ‘Browse’ option with data download facility are provided in this database. Three different modules for mapping and alignment of the query sequences *viz.* BLASTN, SW Align and Mapping are incorporated in AtFusionDB. This database is a head start for exploring the complex and unexplored domain of gene/transcript fusion in plants.

## Introduction

**Figure 1:**
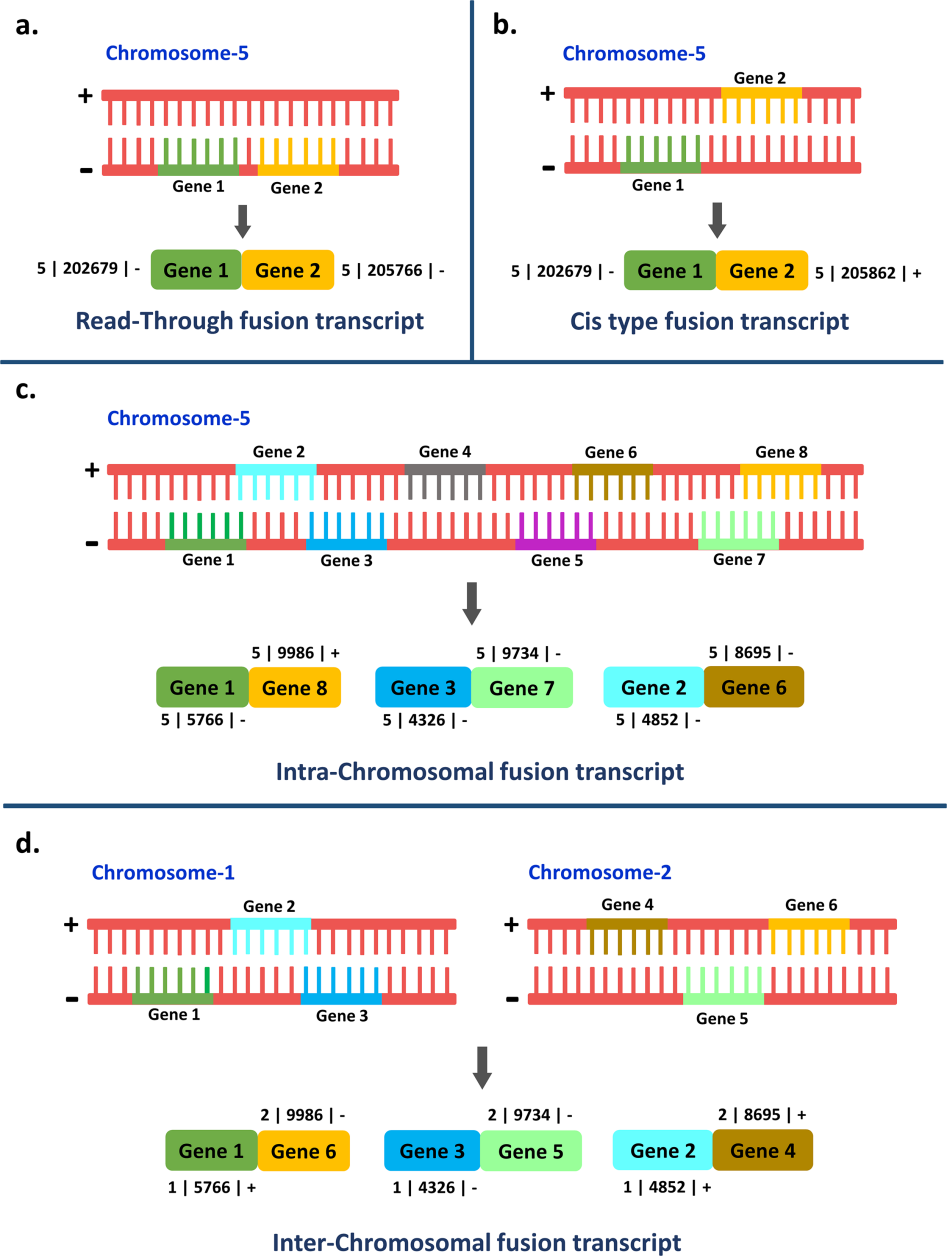
Representation of the four different types of fusion transcripts.

The origin and evolution of new genes are the constant sources of evolutionary renovation and adaptation. Gene duplication, the *de novo* origination of gene, transposition, fission and fusion are the major processes leading to the genesis of new genes (1, 2). Fusion transcripts illustrate an event in which a hybrid RNA is composed of transcripts from two separate genes (3). This can be accomplished by translocation of the original genes at the DNA level or post-transcriptionally during splicing events, and it has been documented in diverse life forms (4). The formation of fusion transcripts can occur either by gene or chromosomal rearrangements (gene fusion at DNA level by translocation, deletion and inversion) or by intergenic RNA cis-splicing and trans-splicing events, i.e. transcript fusion at RNA level (5–7). The fusion transcripts formed post-transcriptionally are more common (8). The splicing event is said to be of ‘cis-type’ when the two exons derived from two neighboring genes transcribe simultaneously or ‘trans-type’ when the two exons originated from two separate premature mRNAs. Nonetheless, both of these aforementioned types of splicing events are mediated by spliceosome complex (9). Read-through fusion transcripts are generated by fusion of the two adjoining individually spliced genes in the same orientation and from the same strand, resembling alternative splicing (10, 11). Likewise, the fusion transcripts that are originated as a result of the hybridization of transcripts from two nearby genes located in opposite strands give rise to cis-acting chimeric transcripts (12). Intra-chromosomal fusion transcripts are generated by fusion of genes or transcripts coming from the same chromosome while inter-chromosomal chimeric transcripts are formed as a result of gene or transcript fusion from different chromosomes (13). The different fusion transcript types *viz.* read-through, cis-acting, intra-chromosomal and inter-chromosomal transcripts have been illustrated in Figure 1.

**Figure 2:**
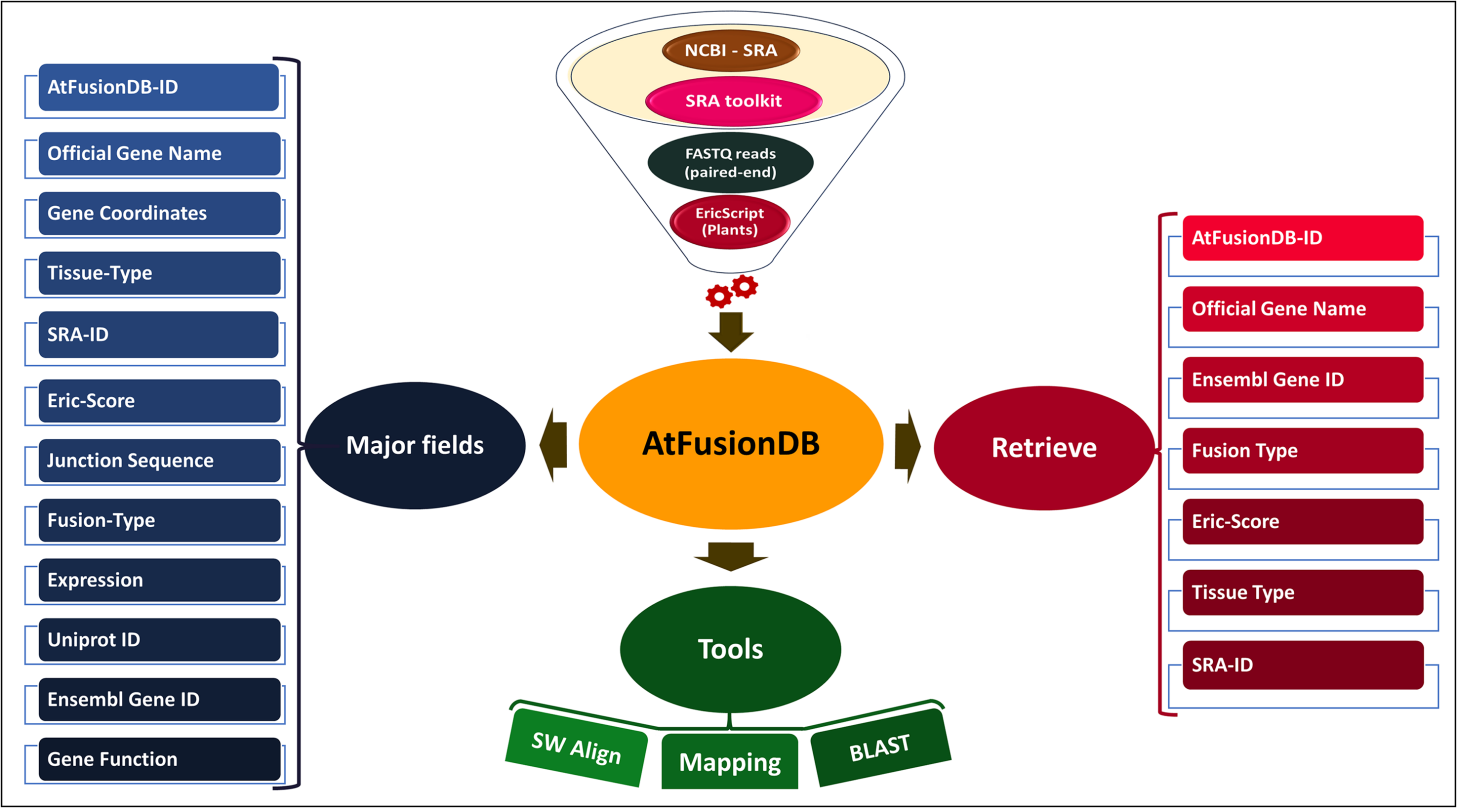
Overall representation of AtFusionDB.

The existence and impact of fusion transcripts at molecular and physiological levels have been studied in eukaryotes including *Drosophila* (14), zebrafish (15), plants (16–18) and humans (19, 20). The role of gene fusion in promoting hematological and solid cancers has been well established in humans (21, 22), and this has paved way to further inquire about their biological relevance in other organisms as well. The very famous and extensively studied BCR-ABL1 fusion transcript is involved in promoting malignancy in the case of chronic myelogenous leukemia (23). These fusion transcripts have been exploited as biomarkers in cancer prediction and targets of molecular therapeutics (24). Chimeric transcripts may either act as long non-coding RNAs or can encode novel chimeric proteins (25), thus can alter cellular signaling and overall functioning in diverse organisms. The emergence of high-throughput technologies has led to the accumulation of enormous sequencing data, which has eased the understanding of the molecular mechanism behind this complex event, and its implications are being attempted to be elucidated in eukaryotic organisms including plants (21). The currently available fusion transcript databases such as ChiTaRS (26), FusionCancer (24), ChimerDB (27), Mitelman Database (28) and FusionHub (29) harbor information related to fusion transcripts reported in human cancers, mouse and flies. Till date, only scarce knowledge about fusion transcripts is available for plants (30–32). A freely available fusion transcript database of plants is currently unavailable to our best of knowledge. In this study, we have developed a database named AtFusionDB which is the plant-exclusive knowledge base for fusion transcripts predicted in the model plant *Arabidopsis thaliana* (the thale cress or mouse-ear cress). The overall structure and major elements of the AtFusionDB are highlighted in Figure 2. Gene fusion is believed to be a major factor for controlling morphology, physiology and phenotypic character in an organism as well as a major contributor for adaptive evolution. Thus, this attempt will unravel new directions for exploring the impact and consequences of gene-fusion events in the plant kingdom and elucidating the significance of shuffling and fusion of transcripts on the physiology of plants.

## Materials and methods

### Data retrieval

We have downloaded all the paired-end RNA-Seq data of *A. thaliana* available at the Sequence Read Archive (SRA) of NCBI (https://www.ncbi.nlm.nih.gov/sra). SRA data were further converted into the FASTQ format using ‘fastq-dump’ utility of SRA Toolkit version 2.8.2-1 (https://www.ncbi.nlm.nih.gov/sra/docs/toolkitsoft/).

### Identification of fusion transcripts

The FASTQ files of paired-end RNA-Seq run obtained from the previous step were given as an input to ‘EricScript-Plants’ (https://github.com/asherkhb/EricScript-Plants) for the identification of fusion transcripts in *A. thaliana*. EricScript-Plants is a modified version of EricScript (33) to work for all the plant species available at Ensembl Plants (34). This version of EricScript was downloaded using the command ‘git clone’ (https://github.com/asherkhb/EricScript-Plants.git). EricScript is a freely available software package (https://sites.google.com/site/bioericscript) for the identification of fusion transcripts from paired-end RNA-Seq data sets. It is developed in Practical Extraction and Reporting Language (PERL) and requires several other dependencies, i.e. R (http://cran.r-project.org/), ada package (http://cran.r-project.org/web/packages/ada/index.html), BWA (35), SAMtools version >0.1.17 (36), bedtools version >2.15 (37), BLAT (38) and seqtk (https://github.com/lh3/seqtk). The major limitation of this script is the use of transcriptome instead of a reference genome for the mapping of sequencing reads. The output of the EricScript-Plants reports the candidate fusions in two tab-delimited files; the first file (e.g. samplename.results.total.tsv) contains all the identified fusions, whereas the other file (e.g. samplename.results.filtered.tsv) reports the fusions with ‘EricScore’ >0.5. In a quality-filtering approach, EricScript exploits three different scores *viz.* genuine junction score, edge score and uniformity score. Using an AdaBoost qualifier (39), these three scores are unified into a single score, called ‘EricScore’, which assigns each candidate fusion a probability score of ‘well’ pattern, and thus classifying all the fusions for discriminating between real transcripts and false-positive events (33). The value of ‘EricScore’ determines the probability of fusion transcripts to be real, with the score ranging from 0.01 to 0.99. The transcripts with the highest EricScore represents the highest possibility to be fusion transcripts. In order to eliminate the false positives, we have only considered the fusion transcripts whose ‘EricScore’ values were >0.5 (e.g. results in the file samplename.results.filtered.tsv). The detailed explanation of the EricScript output files is given at the web page (https://sites.google.com/site/bioericscript/).

The Bioconductor (https://www.bioconductor.org/) packages, SRAdb (40) and GEOmetadb (41) were utilized for the retrieval of tissue-type information of RNA-Seq samples analyzed in this study and combined with each entry of AtFusionDB.

### AtFusionDB web interface development

After the compilation of all the information, AtFusionDB web interface was developed using Hypertext Mark-up Language, Cascading Style Sheets, Structured Query Language, Java scripting language, PERL and Hypertext Preprocessor on Apache Hypertext Transfer Protocol server. The gene coordinates of each individual gene were prepared using the chromosome no., breakpoint and strand sense information separated by ‘|’ like chromosome no. | breakpoint | strand (e.g. 5 | 25563 | +).

### Database organization

The entire data stored in AtFusionDB are organized at different levels. At the most basic or primary level, the user can search by using simple keywords such as ‘gene name’, ‘chromosome’, ‘tissue’, ‘fusion-name’, ‘SRA-ID’, ‘AtFusionDB-ID’ etc. as per the requirement. The information will be displayed in tabular form according to the number of display fields selected by the user. The secondary data can be accessed to gain further information on sequencing experiments and detailed information on fusion transcripts by clicking on the hyperlinks of ‘SRA-ID’ and ‘fusion’ on search result pages, respectively. At the tertiary level, additional information about the contributing genes giving rise to chimeric transcripts can be accessed by clicking on the hyperlinks of the UniProt ID(s) and EnsemblPlants ID(s) on the fusion information page. We have made efforts to make the database easy and convenient to access and fetch the information supplemented with downloadable links.

### AtFusionDB web interface features

AtFusionDB provides the two user-friendly ‘Search’ options *viz.* ‘simple’ and ‘advanced’ for searching fusion transcript information by using different types of keywords. The ‘Simple Search’ option facilitates the user to fetch fusion transcript information by providing different search terms like gene name, chromosome number, tissue etc. To provide flexibility, two options i.e. ‘containing’ and ‘exact’ have been incorporated for search terms. This option also facilitates the user to select the fields to be displayed. The ‘Advanced Search’ option provides the facility to make the user-built query using up to 11 different combinations of keywords. The keywords (e.g. fusion, tissue, chromosomes, genes, ‘EricScore’ etc.) can be defined to be included together or searched alternatively or excluded using ‘add’ and ‘remove facility’. The conditional operators *viz.* ‘=’, ‘Like’ and ‘!=’ and two logical operators ‘OR’ and ‘AND’ can be used as per the need of the users. For convenient browsing, ‘Browse’ section is also available for the user. This section enables the user to browse the database by the following categories: fusion type, tissue type, ‘EricScore’ range, chromosome and frequency. The frequency of occurrence of fusion transcripts can be further browsed with respect to the tissue type, condition and fusion type separately.

**Table 1:** A list of the most frequently occurring fusion transcripts on the basis of fusion type and tissue type

**Fusion type**			
**Cis (5168)**	**Read-through (9313)**	**Intra-chromosomal (16 853)**	**Inter-chromosomal (51 635)**
AT1G66900_AT1G66910 (67) AT1G29920_AT1G29930 (65) AT5G19770_AT5G19780 (57) AT1G29930_AT1G29920 (56)	ATCG00480_ATCG00470 (268) ATCG00810_ATCG00800 (184) AT3G59330_AT3G59320 (131) ATCG00580_ATCG00570 (115)	AT2G07725_AT2G07715 (95) ATCG00065_ATCG01230 (82) AT1G07940_AT1G07920 (80) AT2G36070_AT2G20510 (57)	AT1G07590_AT5G10100 (102) AT4G14960_AT1G50010 (95) AT5G38410_AT1G67090 (88) AT2G45330_AT5G23600 (78)
**Tissue type**			
**Seed (8608)**	**Seedling (36 530)**	**Leaf (7126)**	**Stem (831)**
AT4G11845_AT4G11840 (15) AT4G14960_AT1G50010 (11) AT1G28660_AT1G28670 (9) AT2G15890_AT2G15880 (8)	ATCG00480_ATCG00470 (134) AT5G38410_AT1G67090 (76) ATCG00810_ATCG00800 (74) ATCG00580_ATCG00570 (68)	ATCG00480_ATCG00470 (26) AT1G66900_AT1G66910 (22) ATCG00810_ATCG00800 (18) AT3G59330_AT3G59320 (16)	ATCG00480_ATCG00470 (9) AT1G29920_AT1G29930 (7) AT1G29910_AT1G29920 (7) AT5G38410_AT1G67090 (6)
**Root (8402)**	**Flower and floral parts (4176)**	**Whole plant (11 514)**	**Other (2910)**
AT2G07725_AT2G07715 (33) ATCG00480_ATCG00470 (21) AT4G27090_AT5G10100 (19) AT4G18730_AT5G10100 (16)	AT4G35450_AT4G35460 (7) AT4G25290_AT4G25280 (7) ATCG00810_ATCG00800 (6) ATCG00190_ATCG00180 (6)	AT1G07590_AT5G10100 (88) ATCG00480_ATCG00470 (59) ATCG00810_ATCG00800 (52) AT3G02080_AT5G10100 (46)	AT3G59330_AT3G59320 (12) AT4G14960_AT1G50010 (11) AT3G61780_AT5G28400 (10) ATCG00580_ATCG00570 (9)

**Figure 3:**
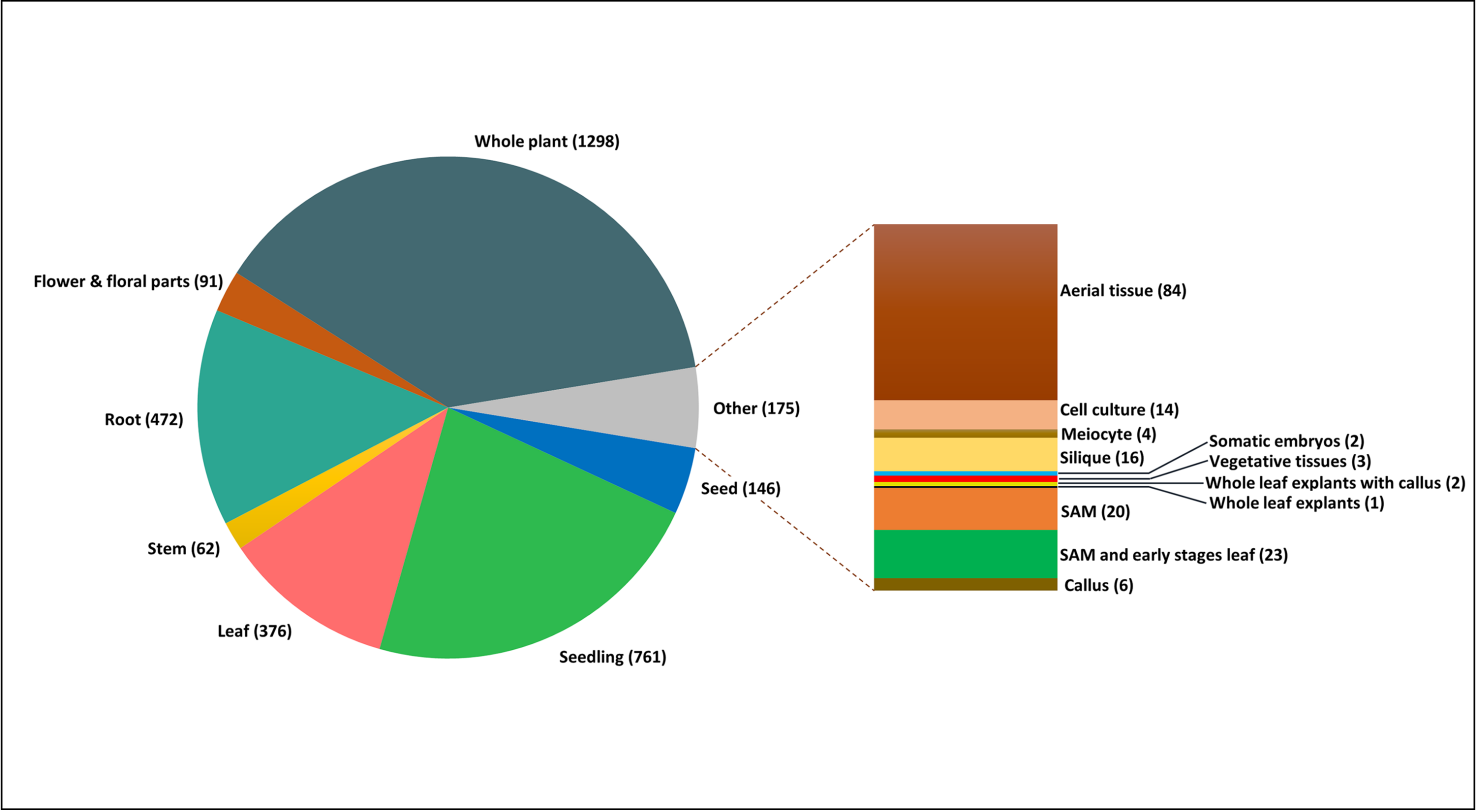
Tissue-wise distribution of total 3520 experimental samples analysed for incorporation into AtFusionDB.

The ‘Tools’ section of AtFusionDB facilitates the user to extract useful information regarding fusion transcript by providing input query to the following tools: ‘SW Align’, ‘Mapping’ and ‘BLAST’ (42). ‘SW Align’ allows the user to align their query sequence with the fusion transcript junction sequences available in AtFusionDB database. This option helps the user to identify and characterize their sequence of interest. Here, we have incorporated ‘WATER’ utility of EMBOSS-6.6.0 package, following the Smith–Waterman Algorithm (43). ‘Mapping’ option facilitates the user to map all the fusion junction sequences from AtFusionDB database to the gene sequences as query provided by the user and only those sequences from AtFusionDB matching 100% with the query sequences are displayed. This module is useful for the detection of fusion transcripts in newly assembled genome drafts or novel gene sequences. In this module, we have incorporated BLASTN (44) option of the BLAST software package. ‘BLAST’ module is helpful to find the regions of similarity between the user input FASTA sequences and AtFusionDB database sequences using BLASTN with the option to change Expect value (E value). The respective ID(s) of sequences from AtFusionDB producing significant alignments with the query sequences are further hyperlinked to display their detailed information.

**Table 2:** Condition-wise study of frequently occurring fusion transcripts

**Condition**	**Number of samples**	**Samples having fusion**	**Ensembl gene1_gene2**	**Fusion type**	**Tissue type**
Wild	138	6	AT1G04820_AT4G14960	Inter-chromosomal	14-day-old seedling, root, rosette leaf, seedling and silique
		6	AT2G36070_AT2G20510	Intra-chromosomal	14-day-old seedling, anther, hypocotyl, seed and seedling
		6	AT4G11845_AT4G11840	Read-through	14-day-old seedling, root, shoot, cotyledon, whole leaf explants with callus, whole plant and whole seedling
		6	ATCG00820_ATCG00810	Read-through	Callus, cell culture, root, rosette leaf and seedling
		7	AT4G30220_AT2G14285	Inter-chromosomal	Anther and rosette leaf
		7	ATCG00480_ATCG00470	Read-through	14-day-old seedling, cell culture, hypocotyl, Rosette leaf, seedling, shoot and cotyledon
Dark-adapted	5	2	ATCG00350_ATCG00340	Read-through	Cell culture and seedling
		2	ATCG00570_ATCG00560	Read-through	Cell culture
		2	ATCG00790_ATCG00780	Read-through	Cell culture
		2	ATCG00810_ATCG00800	Read-through	Cell culture and seedling
		2	ATCG00820_ATCG00810	Read-through	Cell culture
		2	ATCG01120_ATCG01110	Read-through	Cell culture and seedling
		3	ATCG00130_ATCG00120	Read-through	Cell culture and seedling
Drought stress	74	4	AT2G05070_AT2G05100	Read-through	Leaf and rosette leaf
		4	AT3G02065_AT3G02070	Read-through	Rosette leaf
		4	AT3G59330_AT3G59320	Read-through	Rosette leaf
		4	AT3G62310_AT2G47250	Inter-chromosomal	Rosette leaf
		4	AT4G08480_AT4G08470	Read-through	Rosette leaf
		5	AT1G60560_AT1G60590	Intra-chromosomal	Rosette leaf
Heat stress	141	12	ATCG00270_ATCG00280	Read-through	Aerial seedling, flower bud and seedling
		15	AT1G73320_AT1G73310	Read-through	Aerial seedling, flower bud and root
		16	AT3G09162_AT3G09160	Read-through	Aerial seedling, rosette leaf and seedling
		17	ATCG00810_ATCG00800	Read-through	Aerial seedling and flower bud
		18	AT3G59330_AT3G59320	Read-through	Aerial seedling, flower bud, Shoot Apical Meristem (SAM) and early stages leaf and seedling
		28	ATCG00480_ATCG00470	Read-through	Aerial seedling, seedling and whole plant
Oxidative stress	3	2	AT1G53670_AT1G53680	Read-through	Root
		2	AT3G25597_AT3G25600	Read-through	Root
		2	AT4G24413_AT4G24410	Read-through	Root
		2	AT5G56720_AT5G56730	Cis	Root
		2	ATCG00340_ATCG00330	Read-through	Root
		2	ATMG00410_AT2G07707	Inter-chromosomal	Root
		2	ATMG00600_AT2G07648	Inter-chromosomal	Root
		3	AT5G38344_AT5G38350	Read-through	Root
Nematode *Heterodera schachtii* infection	4	2	AT1G23870_AT1G23880	Read-through	Root
		2	AT1G29470_AT3G61260	Inter-chromosomal	Root
		2	AT5G24520_AT5G24510	Read-through	Root
*Pseudomonas aeruginosa* infection	16	3	AT1G76690_AT1G76680	Read-through	Root and shoot
		3	AT2G04100_AT2G04090	Read-through	Root and shoot
		3	AT2G05100_AT2G05070	Read-through	Shoot
		3	AT2G47110_AT3G52590	Inter-chromosomal	Root and shoot
Turnip crinkle virus infection	6	2	AT1G72610_AT1G29920	Intra-chromosomal	Leaf
		2	AT3G12120_AT2G34420	Inter-chromosomal	Leaf
		2	AT3G26395_AT5G27730	Inter-chromosomal	Leaf
		2	AT3G59330_AT3G59320	Read-through	Leaf
		2	AT4G01590_AT4G35685	Intra-chromosomal	Leaf
		2	AT4G15000_AT1G29910	Inter-chromosomal	Leaf
		2	AT5G19770_AT5G19780	Cis	Leaf
		2	ATCG00350_ATCG00340	Read-through	Leaf
		2	ATCG00480_ATCG00470	Read-through	Leaf
		2	ATCG00810_ATCG00800	Read-through	Leaf
Spaceflight grown	139	8	ATCG00480_ATCG00470	Read-through	Root
		9	AT2G25210_AT4G31985	Inter-chromosomal	Root
		9	AT4G27090_AT5G10100	Inter-chromosomal	Root
		13	AT2G07725_AT2G07715	Intra-chromosomal	Root
Nutrient deficient	22	4	AT1G26250_AT1G26255	Cis	Root
		4	AT4G24413_AT4G24410	Read-through	Root
		6	AT2G27010_AT2G27000	Read-through	Root
Abscisic acid treated	12	3	AT2G41200_AT2G41210	Read-through	Seedling
		3	AT5G22794_AT1G49940	Inter-chromosomal	Seedling
		3	ATCG00810_ATCG00800	Read-through	Leaf and seedling

The ‘Method’ section explains the pipeline opted for the identification of fusion transcripts. The ‘Statistics’ page graphically represents the total and unique fusion transcripts incorporated in AtFusionDB on the basis of fusion and tissue types. The user can also visualize a pie chart depicting the distribution of unique and common fusion transcripts found in SRA samples. ‘Help/Guide’ section is useful for the user to understand AtFusionDB database and use it effectively.

### Results and discussion

We have downloaded and analyzed a total of 4697 paired-end RNA-Seq data sets of *A. thaliana*. We could not found the fusion transcripts in 1036 samples because of different logistic reasons (e.g. the quality of data, the quantity of data, the absence of fusion transcripts etc.). Out of remaining 3661 samples, 141 samples have only the fusion transcripts with low EricScore (e.g. <0.5), not considered for further analyses. Finally, the most probable fusion transcripts (with EricScore >0.5) from 3520 samples were incorporated in AtFusionDB database. These 82 969 fusion transcripts with ‘EricScore’ >0.5 were considered for further data processing and refinement in AtFusionDB. Altogether, 17 181 genes were involved in fusion transcript generation. Rank-wise distribution of total predicted fusion transcripts in accordance with ‘EricScore’ range has been listed in the table available at the ‘Statistics’ section of the database.

A total of 82 969 fusion transcript entries of AtFusionDB are represented by 71 920 unique fusion transcripts. A total of 41 838 fusion transcripts were nonrecurrent and found only in one RNA-Seq sample. However, numerous transcripts were observed to be common in two or more than two samples that is graphically represented in the ‘Statistics’ section of AtFusionDB. The fusion transcripts (total and unique) were also categorized and distributed on the basis of their aforementioned fusion types. It was noticed that inter-chromosomal transcripts were the most abundant and cis-acting transcripts were least in number. The graphical representation showing their distribution has been provided in the Statistics section of AtFusionDB. The three most frequently occurring (e.g. recurrent) fusion transcripts in all analyzed samples were ATPB_ATPE, RPL22_RPS3 and PSBE_PSBF. All of these transcripts were of read-through type and originating from chloroplast. Multifold expression of these specific fusion transcripts in contrast to the significantly low expression of their individual contributing genes indicates that they might have a distinct role in governing cellular dynamics.

The tissue-wise study of the total fusion transcript entries, as well as unique chimeric transcripts, was also carried out on different tissues and developmental stages in the life cycle of *A. thaliana*. All the 3520 samples predicted with reliable fusion transcripts were distributed on the basis of their developmental stages and tissue origin. It was observed that most chimeric transcripts were derived from seedling, seed, root and whole plant RNA samples. It was noted that few genes commonly contributing in chimeric transcripts generation in the majority of tissue types were elongation factor 1-alpha 3/4 (A1_A1), chloroplastic ATP synthase subunit beta and ATP synthase epsilon chain (ATPB_ATPE) and Chlorophyll a-b binding protein 3 (LHCB1.1_LHCB1.3) and Tubulin alpha chain and Tubulin alpha-2 chain (TUBA6_TUBA4). The most frequently occurring fusion transcripts on the basis of fusion type and tissue origin along with their respective frequencies are shown in Table 1. The tissue-wise distribution of SRA samples is graphically represented in Figure 3. The samples were also categorized on the basis of different experimental conditions of abiotic and biotic stresses together with their respective frequency as demonstrated in Table 2.

By comparing fusion transcripts from the study done on rice by Zhang and his team in 2010 (16), we found 31 fusion-contributing genes from AtFusionDB homologous to fusion-contributing genes in rice (Supplementary Data Table 1.1). Further, we also found two fusion genes *viz.* AK101547_AK121590 and AK121590_AK101547 from rice that was homologous to 18 fusion genes in our database (Supplementary Data Table 1.2). Similar comparative studies were also performed in *Nicotiana tabacum* (tobacco) and it was observed that 35 different genes from tobacco were homologous to 10 fusion contributing genes from AtFusionDB. It was noticed that the genes expressing ribosomal proteins, tubulin and glyceraldehyde-3 phosphate dehydrogenase were common in all three plants considered for the study, thereby indicating their vital roles as fusion transcripts in governing the physiology of plants. The BLAST data results and gene list supporting homology studies along with their respective functions have been provided in the supplementary files (Supplementary Data Tables 1.1, 1.3, 1.4 and 1.5). Thus, our study has confirmed the previous reports of the existence of chimeric transcripts in rice and also indicates the significance of these novel fusion transcripts in other plants as well.

Despite the continuous efforts from researchers for understanding the origin of life, the evolution of genes, genomes and organisms, innumerable questions related to the birth and evolution of genes, which are the structural and functional unit of life, still remains unanswered. However, the rise of Big Data Era has provided burgeons of sequencing data that can be exploited for a better understanding of diverse mechanisms of origin of new genes and the impact of fusion of genes on each and every aspect of growth, development, physiology and adaptive evolution of the parental organisms as well as their progenies. Although a plethora of gene fusion transcripts has been predicted, an in-depth study, validation and functional characterization of the transcripts together with their encoded products are yet to be accomplished. AtFusionDB is the first attempt to gather and store information related to fusion transcripts in plants. This database will make it easy to explore the significance of gene/transcript fusion in plants. It will prove to be beneficial for the biologists in gaining knowledge of this rarely explored domain in the plant kingdom.

## Authors contributions

D.D. and A.S. developed the web interface of the database. D.D., A.S., S.Z. and S.K. collected and compiled the data and performed the analysis. S.Z. and S.K. wrote the manuscript. S.K. conceived the idea and coordinated the project.

## Supplementary Material

Supplementary Data

## Notes

**Database URL**: http://www.nipgr.res.in/AtFusionDB

## References

[1] Epstein C.J. (1971) Evolution by gene duplication. * Am. J. Hum. Genet.* 23, 541.

[2] Williford A. and Betrán E. (2013) Gene Fusion. In: *eLS*. John Wiley & Sons, Ltd, Chichester, UK.

[3] Kumar S., Razzaq S.K., Duy Vo A.D. et al. (2016) Identifying fusion transcripts using next generation sequencing advanced review *Wiley Interdiscip. Rev. RNA*. 7, 811–823. 27485475 10.1002/wrna.1382 PMC5065767

[4] Kaessmann H. (2010) Origins, evolution, and phenotypic impact of new genes. *Genome Res.*, 20, 1313–1326. 20651121 10.1101/gr.101386.109 PMC2945180

[5] Frenkel-Morgenstern M., Lacroix V., Ezkurdia I. et al. (2012) Chimeras taking shape: potential functions of proteins encoded by chimeric RNA transcripts. *Genome Res.*, 22, 1231–1242. 22588898 10.1101/gr.130062.111 PMC3396365

[6] Qin F., Song Z., Babiceanu M. et al. (2015) Discovery of CTCF-sensitive cis-spliced fusion RNAs between adjacent genes in human prostate cells. *PLoS Genet.*, 11, e1005001. 25658338 10.1371/journal.pgen.1005001 PMC4450057

[7] Li H., Wang J., Ma X. et al. (2009) Gene fusions and RNA trans-splicing in normal and neoplastic human cells. *Cell Cycle*, 8, 218–222. 19158498 10.4161/cc.8.2.7358

[8] Latysheva N.S. and Babu M.M. (2016) Discovering and understanding oncogenic gene fusions through data intensive computational approaches. *Nucleic Acids Res.*, 44, 4487–4503. 27105842 10.1093/nar/gkw282 PMC4889949

[9] Di Segni G., Gastaldi S. and Tocchini-Valentini G.P. (2008) Cis- and trans-splicing of mRNAs mediated by tRNA sequences in eukaryotic cells. *Proc. Natl. Acad. Sci. USA*, 105, 6864–6869. 18458335 10.1073/pnas.0800420105 PMC2383978

[10] Nacu S., Yuan W., Kan Z. et al. (2011) Deep RNA sequencing analysis of readthrough gene fusions in human prostate adenocarcinoma and reference samples. *BMC Med. Genomics*, 4, 11. 21261984 10.1186/1755-8794-4-11 PMC3041646

[11] Varley K.E., Gertz J., Roberts B.S. et al. (2014) Recurrent read-through fusion transcripts in breast cancer. *Breast Cancer Res. Treat.*, 146, 287–297. 24929677 10.1007/s10549-014-3019-2 PMC4085473

[12] Zhang Y., Gong M., Yuan H. et al. (2012) Chimeric transcript generated by cis-splicing of adjacent genes regulates prostate cancer cell proliferation. *Cancer Discov.*, 2, 598–607. 22719019 10.1158/2159-8290.CD-12-0042

[13] Maher C.A., Kumar-Sinha C., Cao X. et al. (2009) Transcriptome sequencing to detect gene fusions in cancer. *Nature*, 458, 97–101. 19136943 10.1038/nature07638 PMC2725402

[14] Rogers R.L., Bedford T., Lyons A.M. et al. (2010) Adaptive impact of the chimeric gene Quetzalcoatl in *Drosophila* melanogaster. *Proc. Natl. Acad. Sci. USA*, 107, 10943–10948. 20534482 10.1073/pnas.1006503107 PMC2890713

[15] Fu B., Chen M., Zou M. et al. (2010) The rapid generation of chimerical genes expanding protein diversity in zebrafish. *BMC Genomics*, 11, 657. 21106061 10.1186/1471-2164-11-657 PMC3091775

[16] Zhang G., Guo G., Hu X. et al. (2010) Deep RNA sequencing at single base-pair resolution reveals high complexity of the rice transcriptome. *Genome Res.*, 20, 646–654. 20305017 10.1101/gr.100677.109 PMC2860166

[17] Koller B., Fromm H., Galun E. et al. (1987) Evidence for *in vivo* trans splicing of pre-mRNAs in tobacco chloroplasts. *Cell*, 48, 111–119. 3791410 10.1016/0092-8674(87)90361-8

[18] Kawasaki T., Okumura S., Kishimoto N. et al. (1999) RNA maturation of the rice SPK gene may involve trans-splicing. *Plant J.*, 18, 625–632. 10417713 10.1046/j.1365-313x.1999.00493.x

[19] Edwards P.A. (2010) Fusion genes and chromosome translocations in the common epithelial cancers. *J. Pathol.*, 220, 244–254. 19921709 10.1002/path.2632

[20] Mertens F., Johansson B., Fioretos T. et al. (2015) The emerging complexity of gene fusions in cancer. *Nat. Rev. Cancer*, 15, 371–381. 25998716 10.1038/nrc3947

[21] Annala M.J., Parker B.C., Zhang W. et al. (2013) Fusion genes and their discovery using high throughput sequencing. *Cancer Lett.*, 340, 192–200. 23376639 10.1016/j.canlet.2013.01.011 PMC3675181

[22] Nothwang H.G., Kim H.G., Aoki J. et al. (2001) Functional hemizygosity of PAFAH1B3 due to a PAFAH1B3-CLK2 fusion gene in a female with mental retardation, ataxia and atrophy of the brain. *Hum. Mol. Genet.*, 10, 797–806. 11285245 10.1093/hmg/10.8.797

[23] Shtivelman E., Lifshitz B., Gale R.P. et al. Fused transcript of abl and bcr genes in chronic myelogenous leukaemia. *Nature*, 315, 550–554. 2989692 10.1038/315550a0

[24] Wang Y., Wu N., Liu J. et al. (2015) FusionCancer: a database of cancer fusion genes derived from RNA-seq data. *Diagn. Pathol.*, 10, 131. 26215638 10.1186/s13000-015-0310-4 PMC4517624

[25] Jia Y., Xie Z. and Li H. (2016) Intergenically spliced chimeric RNAs in cancer. *Trends Cancer*, 2, 475–484. 28210711 10.1016/j.trecan.2016.07.006 PMC5305119

[26] Gorohovski A., Tagore S., Palande V. et al. (2017) ChiTaRS-3.1—the enhanced chimeric transcripts and RNA-seq database matched with protein–protein interactions. *Nucleic Acids Res.*, 45, D790–D795. 27899596 10.1093/nar/gkw1127 PMC5210585

[27] Kim N., Kim P., Nam S. et al. (2006) ChimerDB—a knowledgebase for fusion sequences. *Nucleic Acids Res.*, 34, D21–D24. 16381848 10.1093/nar/gkj019 PMC1347382

[28] Mitelman F., Johansson B. and Mertens F. (2007) The impact of translocations and gene fusions on cancer causation. *Nat. Rev. Cancer*, 7, 233–245. 17361217 10.1038/nrc2091

[29] Panigrahi P., Jere A. and Anamika K. (2018) FusionHub: a unified web platform for annotation and visualization of gene fusion events in human cancer. *PLoS One*, 13, e0196588. 29715310 10.1371/journal.pone.0196588 PMC5929557

[30] Chen J.J. (1997) A gene fusion at a homeobox locus: alterations in leaf shape and implications for morphological evolution. *Plant Cell*, 9, 1289–1304. 9286107 10.1105/tpc.9.8.1289 PMC156998

[31] Wang W., Zheng H., Fan C. et al. (2006) High rate of chimeric gene origination by retroposition in plant genomes. *Plant Cell*, 18, 1791–1802. 16829590 10.1105/tpc.106.041905 PMC1533979

[32] Chen C.J., Liu Q., Zhang Y.C. et al. (2011) Genome-wide discovery and analysis of microRNAs and other small RNAs from rice embryogenic callus. *RNA Biol.*, 8, 538–547. 21525786 10.4161/rna.8.3.15199

[33] Benelli M., Pescucci C., Marseglia G. et al. (2012) Discovering chimeric transcripts in paired-end RNA-seq data by using EricScript. *Bioinformatics*, 28, 3232–3239. 23093608 10.1093/bioinformatics/bts617

[34] Bolser D., Staines D.M., Pritchard E. et al. (2016) Ensembl Plants: Integrating Tools for Visualizing, Mining, and Analyzing Plant Genomics Data., *Methods in Molecular Biology (Clifton, N.J.)*, 1374, 115–140. 10.1007/978-1-4939-3167-5_6 26519403

[35] Li H. and Durbin R. (2009) Fast and accurate short read alignment with Burrows–Wheeler transform. *Bioinformatics*, 25, 1754–1760. 19451168 10.1093/bioinformatics/btp324 PMC2705234

[36] Li H., Handsaker B., Wysoker A. et al. (2009) The Sequence Alignment/Map format and SAMtools. *Bioinformatics*, 25, 2078–2079. 19505943 10.1093/bioinformatics/btp352 PMC2723002

[37] Quinlan A.R. and Hall I.M. (2010) BEDTools: a flexible suite of utilities for comparing genomic features. *Bioinformatics*, 26, 841–842. 20110278 10.1093/bioinformatics/btq033 PMC2832824

[38] Kent W.J. (2002) BLAT—the BLAST-like alignment tool. *Genome Res.*, 12, 656–664. 11932250 10.1101/gr.229202 PMC187518

[39] McPherson A., Hormozdiari F., Zayed A. et al. (2011) deFuse: an algorithm for gene fusion discovery in tumor RNA-Seq data. *PLoS Comput. Biol.*, 7, e1001138. 21625565 10.1371/journal.pcbi.1001138 PMC3098195

[40] Zhu Y., Stephens R.M., Meltzer P.S. et al. (2013) SRAdb: query and use public next-generation sequencing data from within R. *BMC Bioinformatics*, 14, 19. 23323543 10.1186/1471-2105-14-19 PMC3560148

[41] Zhu Y., Davis S., Stephens R. et al. (2008) GEOmetadb: powerful alternative search engine for the Gene Expression Omnibus. *Bioinformatics*, 24, 2798–2800. 18842599 10.1093/bioinformatics/btn520 PMC2639278

[42] Altschul S.F., Gish W., Miller W. et al. (1990) Basic local alignment search tool. *J. Mol. Biol.*, 215, 403–410. 2231712 10.1016/S0022-2836(05)80360-2

[43] Smith T.F. and Waterman M.S. (1981) Identification of common molecular subsequences. *J. Mol. Biol.*, 147, 195–197. 7265238 10.1016/0022-2836(81)90087-5

[44] Chen Y., Ye W., Zhang Y. et al. (2015) High speed BLASTN: an accelerated MegaBLAST search tool. *Nucleic Acids Res.*, 43, 7762–7768. 26250111 10.1093/nar/gkv784 PMC4652774

